# Exploration of Effective Substance Use Relapse Prevention Programmes for the Youth: An Integrative Literature Review

**DOI:** 10.1111/jpm.13144

**Published:** 2024-12-05

**Authors:** Wada Gaolaolwe, Miriam Mmamphamo Moagi, Gaotswake Patience Kovane, Leepile Sehularo

**Affiliations:** ^1^ NuMIQ Research Focus Area Faculty of Health Sciences North‐West University South Africa; ^2^ Department of Health Sciences, School of Nursing University of Botswana Gaborone Botswana; ^3^ Nursing Department University of Limpopo Sovenga South Africa; ^4^ Lifestyle Diseases Research Focus Area, Faculty of Health Sciences North‐West University Mafikeng South Africa

**Keywords:** check‐ups, prevention, programme, relapse, substance use

## Abstract

**Introduction:**

Youth substance use has been associated with recurrent episodes of substance misuse. A large body of research has shown that relapsing into substance use is still a significant obstacle that prevents people who use substances and want to stop from recovering and abstaining. The objective of this evaluation was to locate, compile and summarise the results of all pertinent research on youth substance use relapse prevention programmes.

**Method:**

An integrative literature review (ILR) was conducted, guided by Whittemore and Knafl (*Journal of Advanced Nursing*, 2005, **52**, 546), following a step‐by‐step guide for conducting an ILR. The PRISMA guidelines were used in the selection process.

**Results:**

Twenty‐three papers met the eligibility criteria, and three were added from grey literature. Three themes identified in the studies included in the review: Continuing care, technology‐mediated recovery management interventions and relapse prevention through developmentally engaging activities.

**Discussion:**

The review identified that a successful substance use relapse prevention programme for the youth requires continued care following discharge from hospital treatment. The care encompasses posttreatment check‐ups, assessments and linkages to treatment in which a suspected/potential relapse is referred back to treatment to prevent its severity or occurrence.

**Summary**

- What is known on the subject?
  ○ Substance users often relapse back to substances after a period of abstinence and the problem can be abated through posttreatment check‐ups.
  ○ Tracking, assessment and linkages to treatment are important components of a relapse prevention programme.
- What the paper adds to existing knowledge?
  ○ The paper draws together the existing literature on available programmes that are aimed at preventing substance use relapse among the youth.
  ○ In addition to tracking, assessment and linkages, a robust relapse prevention programme for the youth must embrace technology for personalised engagement, monitoring and feedback.
  ○ Youth psychoeducation must be delivered creatively to alleviate the cognitive dissonance that some youth experience. Psychoeducation becomes appealing to young people if it covers some key neuroscience principles that build their understanding of brain functions and the impact of substances on the brain.
- What are the implications for practice?
  ○ Mental health nurses need to understand how to effectively assist the youth with substance use relapse problems to achieve sustained abstinence and sobriety through continued posttreatment care and linkages to treatment.

## 1 Introduction

The youth's relapse into substance use is extremely distressing (You et al. 2020). Young people are going through a transitional period in their development where they discover who they are, develop new abilities, form relationships with their peers and cultivate a positive body image. These changes are not always easy to make, though, and sometimes they include risk‐taking behaviours like substance use when the brain is still developing (Arora et al. 2015; Ryan et al. 2019). Youth is the period of life when one is young, which is a period between childhood and maturity (adulthood). It is often defined in terms of the ages of leaving education compulsorily to when the person gets their first job (UNESCO 2019; Manyanda et al. 2021). Youth substance use is the unsafe indulgence in licit or illicit substances by young people for mood‐altering purposes in a way that is a potential threat to their well‐being, the well‐being of their families and even the well‐being of their communities (Mhaka‐Mutepfa 2021). Youth substance usage increases the likelihood of developing a dependency on the substances in the future and relapsing into use (Kabisa et al. 2021; More et al. 2017). Considering this, youth substance use is characterised by recurrent relapses. There is abundant evidence that for those who use substances and want to stop, relapse is still a significant obstacle that prevents them from recovering and staying sober (Appiah 2022; Furzer et al. 2021; Volkow 2020; You et al. 2020). Sixty‐five to eighty‐five per cent of young persons with substance use disorders (SUDs) experience a relapse 12 months after starting treatment (Furzer et al. 2021; Lopes‐Rosa et al. 2017). Substance use treatment has historically raised hopes that it would end the ‘revolving door’ that allowed patients with addiction issues to enter and exit institutions (White and Kelly 2011). Existing research, however, indicates that effective relapse prevention strategies and programmes are necessary and must take into consideration social, cultural, environmental and individual factors to prevent substance use relapse (Appiah et al. 2018; Kabisa et al. 2021; Maikano et al. 2021).

Programmes for preventing substance use relapses are crucial because they help substance users become more in control of their impulses, learn how to curb cravings and avoid relapsing into substance use, build new social skills and postpone gratification (WHO‐UNODC 2020). Research on substance use relapse prevention programmes and recovery trajectories has just lately begun to gather, despite the awareness that young people participate in dangerous substance use with a high rate of relapse. Randomised controlled trials have been used to demonstrate the effectiveness of numerous substance use relapse prevention programmes, many of which have been developed, especially in Western countries, to prevent substance use relapse. These programmes include, among others, web‐based preventative initiatives, recovery management examinations and cognitive behavioural‐based programmes (Karno et al. 2021; Rooke et al. 2013; Scott et al. 2023; Trudeau et al. 2017). In a study conducted in the USA, the effectiveness of an online relapse prevention programme called Navigating my Journey (NmJ) was evaluated among young people aged 13–24. The study found that participants who used this programme experienced increased self‐efficacy and reduced substance use over time. Still in the USA, a study conducted to test the effectiveness of a brief intervention programme known as SBIRT (Screening, Brief Intervention and Referral to Treatment) was effective in reducing the frequency of substance use in young people who were 18 years and older (Karno et al. 2021; Trudeau et al. 2017).

Substance use among young people is also a problem in Australia, where a neuroscience‐based relapse/harm reduction programme was launched with a focus on school‐age adolescents, namely, junior high school pupils (12–15 years old) (Debenham et al. 2020). However, according to reports, this relapse prevention programme's effectiveness is minimal to nonexistent (Debenham et al. 2020). Relapse prevention programmes involving expressive arts are becoming more common in Hong Kong, both in inpatient residential treatment facilities and community‐based counselling centres (Tam, Shik, and Lam 2016). Nevertheless, in contrast to Western nations, no empirical randomised controlled trials have been carried out to determine the efficacy of these SUR programmes (Tam, Shik, and Lam 2016). Substance use relapse prevention programmes are lacking in African nations where access to empirically based treatments is limited (Gouse et al. 2016; Pasche et al. 2015). In Botswana, a middle‐income country in sub‐Saharan Africa, the absence of substance use relapse prevention programmes hinders the effective prevention of substance use relapse. Botswana has limited rehabilitation services, with the primary focus being on inpatient rehabilitation after a relapse. To the best of the researcher's knowledge, there are no specific programmes in place to prevent relapses to substance use after hospital discharge.

Conducting an integrative review related to SUR prevention for the youth in the current study is very important, given the high rate of SURs, the burden they place on society, the setback they impose on recovery successes and the limited understanding of SUR prevention (Kabisa et al. 2021; More et al. 2017). The report from the World Health Organisation (WHO) and the United Nations Office on Drugs and Crime (UNODC) shows young people as a particularly susceptible population in need of specialised care (WHO‐UNODC 2020). The findings of this review will assist mental health nurses in understanding the components of a youth substance use relapse prevention programme and work to reduce substance‐related relapses that impede successful recovery.

### 1.1 Rationale and Aim

Substance use has been a public health concern for decades, especially among the youth with harmful outcomes such as undesirable behaviour, compromised health status, legal and economic implications (Arora et al. 2015; Oladele 2021). Though substance use is common among youth, their relapse following abstinence causes great concern (Gonzales‐Castaneda et al. 2022). However, despite the concern, substance use relapse prevention remains a challenge with literature suggesting a variety of relapse prevention programmes (Arora et al. 2015; You et al. 2020). Thus, this review aimed to identify, synthesise and present the findings of all relevant studies on substance use relapse prevention programme for the youth. The research question for the review was as follows:

- What is stated in the published literature on a substance use relapse prevention programme for the youth?

## 2 Methods

An integrative literature review (ILR) was conducted, guided by Whittemore and Knafl (2005). A step‐by‐step guide to conducting an ILR was followed (Toronto and Remington 2020). This is a guide that was expanded from the previous guidelines for conducting an ILR (Whittemore and Knafl 2005). An ILR allows the inclusion of diverse methodologies to explore different perspectives on the subject matter (Toronto and Remington 2020). Since our review required broad research evidence on substance use relapse (SUR) prevention for the youth, an ILR best served the purpose of our review. Thus, after problem identification, a comprehensive review of the literature was conducted guided by steps suggested by Whittemore and Knafl (2005) as follows:

### 2.1 Problem Identification

The identified problem for this integrative review emanated from study reports and anecdotal notes from experts that identified substance use relapse as a serious concern in Botswana and globally, with a lack of prevention strategies. The review was guided by the following research question: What is stated in the published literature on a substance use relapse prevention programme for the youth?

### 2.2 Literature Search Strategy

The search strategy for this review is discussed under the subheadings below.

#### 2.2.1 Inclusion Criteria

In applying the inclusion criteria, the reviewers systematically examined the literature to critically appraise its quality in terms of its value, reliability and relevance to substance use relapse prevention programme for the youth (Toronto and Remington 2020). The review included literature published in the English language that spanned from 2013 to 2024. The literature used was from diverse methodologies (experimental and nonexperimental studies) that provided different perspectives on the prevention of SUR among the youths. The review also included the grey literature based on the information it yielded in response to the research question.

#### 2.2.2 Exclusion Criteria

The review excluded all articles that were not appropriate in answering the research question, such as studies that did not address substance use relapse prevention programmes or strategies. Moreover, commentaries, letters and editorials were excluded from the review as the review focus was on theoretical, empirical and expert reports on the topic.

#### 2.2.3 Database Searching

The first author (WG) conducted a comprehensive search that captured the most relevant literature. The robustness of the search was enhanced by involving the services of an experienced librarian in reviewing the literature for inclusion and exclusion criteria. The librarian suggested websites and databases that were included in the search. The literature search was conducted in a systematic and comprehensive approach on PubMed, Sabinet African Journals, EBSCOhost and Medline electronic databases. The Boolean operator literature search strategy was used, and it yielded the most appropriate literature for the study (Lubbe, ten Ham‐Baloyi, and Smit 2020). The keywords used for searching the literature were ‘substance OR drug’ AND ‘use OR abuse OR misuse’; ‘relapse prevention OR addiction management’ AND programme OR check‐ups' AND ‘youth OR young adults OR young people’.

**FIGURE 1:**
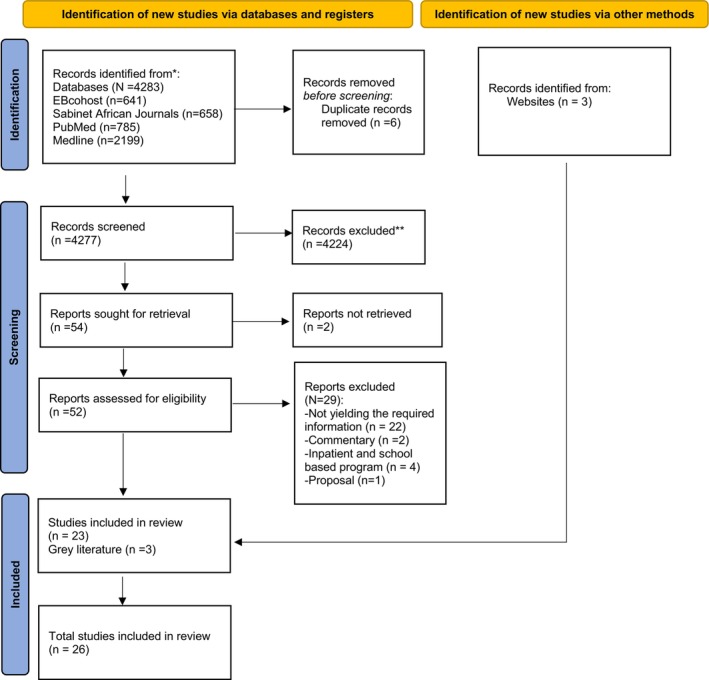
PRISMA flowchart for search strategy.

The literature search resulted in 4283 studies. Then, the researchers embarked on the screening of titles for relevant publications. The researchers excluded duplicated publications and studies that could not be retrieved. Based on the inclusion and exclusion criteria, 4224 publications were excluded from the review. The screening resulted in 52 publications remaining for assessment of abstracts. The researchers assessed and analysed the abstracts to select publications that were directly related to SUR prevention, which further excluded 29 documents. Critical appraisal of the full text was conducted on the 23 remaining publications and 3 documents from the grey literature resulting in reviewing 26 documents. The grey literature consulted was from two websites being, the World Health Organisation (WHO) and the United Nations Office on Drugs and Crime (UNODC) website, and the International Society of Substance Use Professionals (ISSUP) website. The search was conducted from October 2023 to January 2024. The selection process of studies is shown in the Preferred Reporting Items for Systematic Reviews and Data Analysis (PRISMA) (Figure 1).

### 2.3 Data Evaluation

All studies included in the ILR underwent a critical appraisal for quality using the Mixed Methods Appraisal Tool (MMAT) version 2018. Since ILR allows a broad search for evidence, the quality appraisal process enabled researchers to assess empirical studies from diverse methodologies. In the bid to ensure quality results, the reviewers independently conducted a quality appraisal of the studies. The quality ratings of each criterion and the overall score for each study were assessed. However, studies were not excluded from the review based on their methodological quality to avoid inadequate reporting practices and possible exclusion of valuable findings (Lubbe, ten Ham‐Baloyi, and Smit 2020; Whittemore and Knafl 2005). Thus, the researchers were able to identify broad evidence regarding substance use relapse prevention programme for the youth.

### 2.4 Data Synthesis and Analysis

Data analysis involves organising and synthesising data to answer a research question (Polit and Beck 2017). Data analysis for this review was guided by the integrative review process of Whittemore and Knafl (2005). It is during data analysis that the researchers conducted data extraction and data synthesis using content analysis (Lubbe, ten Ham‐Baloyi, and Smit 2020). The researchers developed a data extraction table for the identified articles and captured the author's names (first author), the aim of the study, the methodology used and the findings (Lubbe, ten Ham‐Baloyi, and Smit 2020) (Table 1). During data synthesis, the researchers engaged in a creative process in which interrelated ideas from various studies were clustered to develop a new understanding of substance use relapse prevention (Grove and Gray 2022). The data from primary sources were ordered, coded, categorised and synthesised into a coherent, integrated conclusion about programme for the prevention of substance use relapse (Whittemore and Knafl 2005). The researchers independently reviewed the articles to avoid biases and for consensus on the identified themes and subthemes (Toronto and Remington 2020). Subsequently, this interactive process resulted in the identification of three themes and their subthemes for the review. The identified themes and subthemes are displayed in Table 2.

**TABLE 1:** Appraisal of documents for substance use relapse prevention programmes.

First author	Intervention	Setting	Design	Population	Aim	Data collection/analysis	Major findings	MMAT quality criteria (yes = 1, no = 0, cannot tell = 0)					
								1	2	3	4	5	∑
Bowen et al. (2014)	Participants were assigned randomly to 8 weekly group sessions of cognitive‐behavioural relapse prevention (CBT‐RP), Mindfulness‐based relapse prevention (MBRP) or treatment as usual (TAU‐12 step programme and psychoeducation)	Two‐site, nonprofit chemical dependency treatment agency, Washington, USA	Randomised clinical trial (RCT)	18 years or older with substance use problems	To evaluate the long‐term effectiveness of MBRP in the reduction of relapse compared with RP and treatment as usual (TAU‐12 step programme) during a 12‐month follow‐up period	Intent‐to‐treat analyses were conducted by use of sample size–weighted orthogonal contrasts 2 between MBRP and RP vs. contrast 1 (TAU) and (contrast 2) (MBRP vs. RP)	MBRP and RP had a significantly lower risk of relapse to substance use (SU) and heavy drinking compared to TAU CBT‐RP showed an advantage over MBRP For the 12‐month follow‐up, MBRP participants had significantly fewer days of SU and significantly decreased heavy drinking compared with TAU and RP	1	1	1	1	1	5
Debenham et al. (2020)	Neuroscience‐based, harm minimisation programme	Organisations in Metropolitan Sydney, Australia	A pilot study examining the feasibility of The Illicit programme	Youths at a youth centre and a school	To examine the feasibility of an illicit drug prevention project	Questionnaire/Statistical Package for Social Sciences (SPSS)	The illicit project was good or very good Neuroscience content was interesting, engaging and participants planned to apply the concepts learned in their own lives Programme should find creative ways to alleviate cognitive dissonance in youths Facilitating programme using the Internet	0	1	0	0	1	2
Dennis, Scott, and Laudet (2014)	Reviewing some recent findings related to recovery management	Not applicable	Literature review	Not applicable	Review recent findings related to recovery management	Articles review	Continuing care is important and should be longer, and include therapies like CBT Technological intervention Use of telephone in continuing care and referral to outpatient and self‐care was associated with significantly more abstinent days Recovery management check‐ups for long‐term monitoring and regular check‐ups Use of self‐help or peer support	N/A					
Furzer et al. (2021)	Exercise rehabilitation programme and relapse prevention efficacy, and health outcomes for youth	Australia	Survey	Participants (youths) attending SUD facility	To investigate associations between exercise enjoyment and recovery outcomes for youth undergoing SUD treatment	Questionnaire/R version 31.1.3	Youth who, on average, enjoyed exercise more had higher self‐esteem, perceived physical health, and relapse prevention efficacy than those who enjoyed it less The programme incorporated a range of counselling, case management and support, family therapy, medical reviews and mentoring	1	1	1	1	1	5
Gonzales et al. (2013)	Patient engagement	Los Angeles County, California, USA	Mixed method	Youths in four residential and ten outpatient settings	To explore youth attitudes about recovery‐related needs and important drug‐avoidance behaviours after treatment	Focus group and survey Statistical Program for Social Sciences (SPSS) v20 and ATLAS.ti	Recovery promotion through engaging activities Promoting the use of coping skills for stress management Improve self‐identities Delivering education on harmful consequences of drug abuse	1	1	1	1	1	5
Gonzales et al. (2014)	Exploratory study to examine youth opinions	Outpatient and residential areas in diverse regions of Los Angeles County USA	Exploratory study (mixed method)	Youth aged 12–24 years	To examine youth opinions about the use of text messaging to support recovery behaviours after treatment	Statistical Program for Social Sciences (SPSS) v18 for basic demographic and primary drug information Interviews using eight focus group (ATLAS.ti for analysis)	Most youths in the programme liked the use of text messaging to be reminded to stay on track with recovery Recovery support messages included lifestyle change tips, informational resources, positive appraisal, coping advice, confidence boosters and inspirational encouragement	1	1	1	1	1	5
Gonzales, Douglas Anglin, and Glik (2014)	Project ESQYIR (Educating & Supporting Inquisitive Youth in Recovery)	Los Angeles County, California USA	Randomised, controlled pilot trial of a mobile‐based after care project	Youths in the six community‐based substance abuse programme	To assist youth with recovery and self‐regulating key areas associated with relapse during the initial 3 months after treatment	Statistical Package for Social Sciences (SPSS), version 20.0, and SAS, version 9.3	Mobile aftercare (texting) is vital to engage youth in recovery after substance abuse treatment Texting intervention served as a buffer to substance use relapse Mobile aftercare promotes lifestyle behaviour change	1	1	1	0	1	4
Gonzales‐Castaneda et al. (2022)	Project ESQYIR‐Educating and Supporting in Quisitive Youth In Recovery, utilising text messaging to monitor relapse and recovery processes	Los Angeles USA	Randomised, controlled trial	Youth who completed SUD treatment	To report the impact of a study that measured the impact of an aftercare intervention on primary substance use relapse among youth	Quantitative analysis with a JMP Pro Version 14	Youth constitute the ‘digital natives’ and are facile with technology. So, mobile interventions provide a developmentally and culturally appropriate tool for recovering youth The digital intervention (messaging) was done throughout a 12‐week programme that included monitoring, feedback, education/support and reminders	1	1	1	0	1	4
Guarino et al. (2016)	Check‐In Programme with skills‐based module and self‐management module	Out‐patient methadone maintenance treatment (MMT) programme, New York, USA	Randomised, controlled trial	At least 18 years of age and met DSM criteria for opioid dependence	To evaluate the effectiveness of a web‐based psychosocial programme, i.e., the Therapeutic Education System (TES) delivered to opioid‐dependent individuals	Statistical Program for Social Sciences (SPSS) for quantitative analysis Semistructured interviews used for open‐ended feedback survey items and field notes	Providing clients with psychosocial support outside their formal treatment setting has the potential to enhance treatment retention and reductions in illicit opioid use Delivering intervention content to clients through mobile phone is a feasible approach Participants used the self‐management module more frequently	0	1	1	1	1	4
Haug et al. (2023)	SmartCoach digital (mobile) addiction prevention programme	Swiss cantons of Aargau and Zurich Switzerland	Survey	Young people in secondary than upper secondary school classes	To examine a set of socioeconomic and other predictors of programme participation and programme use with a digital life skills intervention programme	Generalised linear mixed models (GLMMs) to examine predictors of programme use and participation	Generally, almost half of programme participants took part in interactions prompted by the SMS text messaging programme	1	1	1	1	1	5
ISSUP (2020)	Recovery Management and Relapse Prevention	Not applicable	Participant manual	Global community of substance use professionals in diverse sectors, e.g., government, civil society and the private sector	To provide participants with the knowledge and practical skills on substance use relapse and recovery	Training manual	Programmes for SU recovery management have the following elements: Tracking Assessment Linkage Engagement Retention	N/A					
Karno et al. (2021)	Screening, Brief Intervention and Referral to Treatment (SBIRT) Health education session	Mental health centres in Los Angeles County and Ventura County in California, USA	Randomised controlled trial	At least 18 years old with a mental illness diagnosis and heavy drinking or use of cannabis or stimulants in the past 90 days	To establish if over the 6‐ and 12‐month period SBIRT participants would have less frequent heavy drinking and use of stimulants and cannabis To establish the likelihood of abstaining from substances in SBIRT participants relative to participants in the control condition	RStan package and Stan software	Participants on SBIRT were more likely to have less drinking than those in the control arm (exposed to health education only). Thus, screening and brief intervention for unhealthy substance use were effective at reducing the frequency of heavy drinking and stimulant use Use of substances should be identified and brief behavioural intervention offered Substance use screening should be incorporated into the routine mental health intake process	1	1	1	1	1	5
Mitchell et al. (2021)	Participants were assigned either to extended‐release naltrexone (XR‐NTX) or Treatment as usual (TAU)	Mountain Manor Treatment Centre, Baltimore, Maryland, USA	Randomised controlled trial Generalised linear mixed modelling	Youths, ages 15–21 years	To determine self‐reported substance use or relapse to illicit opioid use and other illicit substances such as marijuana, cocaine and alcohol use	Self‐reported 90‐day opioid use Generalised linear mixed modelling (GLiMM) approach	Participants who received XR‐NTX for opioid use disorder (OUD) demonstrated lower retention in treatment than those who did not receive it Use of XR‐NTX as a treatment option for youth motivated to receive it should be considered for relapse prevention	1	1	1	1	1	5
Mpanza, Govender, and Voce (2022)	Explored the perspectives of service providers	KwaZulu‐Natal; South Africa	Qualitative exploratory study design	SUD service providers with at least 1 year in SUD‐related service provision	To gain a full understanding of the aftercare service provision from the perspectives of service providers for people with SUD	Semistructured interviews and focus group discussions NVivo Pro 12 qualitative data analysis software Thematic analysis	There are inconsistencies in the provision of the aftercare services which are already limited Programmes for aftercare services should include relapse prevention Programmes for aftercare activities to involve regular and consistent monitoring, engaging in recreational activities and skills development	1	1	1	1	1	5
NIDA (2018)	A research‐based guide	Not applicable	Guide for effective addiction treatment	A resource document for healthcare providers, family members and other stakeholders in SUD management	A resource for healthcare providers and other stakeholders involved in addressing the problems faced by people in need of treatment for drug abuse or addiction	Not applicable	Substance use programme needs continuous monitoring, as lapses during treatment do occur The programme to incorporate behavioural therapies (individual, family or group counselling) are commonly used forms of substance abuse treatment	N/A					
Scott et al. (2018)	Recovery Management Checkup intervention adapted for primary care settings (RMC‐PC)	State of Illinois USA	Quasi‐experimental study design	Patients who required SUD treatment	To examine whether the RMC intervention could increase the rates of treatment utilisation beyond those obtained with SBIRT only	IBM SPSS, version 23	SBIRT as the usual procedure for screening individuals and referring those needing treatment, an assertive linkage and engagement intervention, the RMC‐PC, has better treatment outcomes in all settings RMC‐PC is a robust linkage model for drug‐using patients in primary care settings	0	0	1	1	1	3
Scott et al. (2023)	Screening, brief intervention and referral to treatment (SBIRT) only or with recovery management check‐ups for primary care (SBIRT+RMC‐PC)	Four primary care sites in Chicago, USA	Randomised controlled trial	People with substance use problems aged 18 years and above	To assess the efficacy of adding RMC‐PC to SBIRT	General linear models with SPSS version 27	The ‘referral to treatment’ component of SBIRT when combined with RMC for patients in primary care settings is highly effective in improving linkages to treatment Repeated check‐ups on longer term treatment is needed	1	1	1	1	1	5
Tam, Shik, and Lam (2016)	Art‐based and cognitive‐behavioural‐based relapse prevention interventions	A Counselling centre for psychotropic substance abusers (CCPSA) in Hong Kong	Two‐group (pre and post) with 6‐month follow‐up experimental design	Young drug abusers aged 15–30 years	To assess the effectiveness of the art‐based and cognitive‐behavioural relapse prevention programmes	Semistructured interviews. NVivo 10 software and thematic analysis	The art‐based relapse prevention group was as effective as the cognitive‐behavioural‐based group	1	1	1	1	1	5
Teko, Goliath, and Abdulla (2023)	Community‐led support groups in SUD recovery Perspectives of the participants	Community support groups in Port Elizabeth in the Eastern Cape, South Africa	Qualitative, interpretive paradigm and an exploratory, descriptive and contextual research design	Young people aged 18–35 years with substance use problems at two community support centres	To explore the value of support groups and the ways in which the support groups can enable SUD sustained recovery from community‐led support groups	Individual semistructured interviews Thematic analysis	Community‐led support groups provide an important platform where the needs of the youths with SUD can be realised. Thus, community‐led support groups can be mobilised and facilitated by trained personnel to strengthen the protective processes of these groups	1	1	1	1	1	5
Trudeau et al. (2017)	Navigating my Journey (NmJ) programme‐teaching, and eliciting evidence‐based protective factors (e.g., problem‐solving) while addressing potential risk factors	Outpatient substance abuse centre. California, USA	Parallel group randomised controlled trial (RCT) Linear mixed modelling approach	Adolescent participants (aged 13–21) with drug and/or alcohol use and completed a detox	To assess if the control condition (those in the NmJ condition) would report higher motivation to reduce substance use and higher self‐efficacy to avoid substance use and improve relapse coping skills	Data collection was online using the software Vovici 6 Enterprise Edition. All analyses were done using GLIMMIX procedure	Participants in the NmJ intervention had improved motivation to reduce substance use and decreased substance use compared to the control group	1	1	1	1	1	5
Van der Westhuizen (2015)	Explores different models of care	Not applicable	Reviewed literature (models)	Not applicable	To link identified models to the specific needs of chemically addicted adolescents	Not specified	Social reintegration is important Motivational interviewing and CBT are important in relapse prevention. The Aftercare programme should not be less than 3 months	N/A					
Wang et al. (2018)	Weekly 10‐session outpatient family‐oriented treatment programme	Outpatient of Kaohsiung Chang Gung Memorial Hospital, Taiwan	Quantitative	Youths with substance use problems (ketamine and stimulant use)	To compare the relapse rates among adolescents with ketamine use and those with stimulant use	Statistical software package SPSS, version 16.0 (SPSS Inc., Chicago, IL, USA)	Youths who recovered from substance use commonly improved or changed their lifestyles Treatment programmes like Motivational Enhancement (MEP) and parental skill training (PST) reduced relapse rates	1	1	1	1	1	5
WHO‐UNODC (2020)	International standards for the treatment of drug use disorders	Not applicable	Not applicable	A guide (revised edition incorporating results of field testing)	To support Member States to develop effective, evidence‐based and ethical treatment for drug use disorders	Not applicable	The following are important in substance use aftercare: Poststabilisation Monitoring Recovery education and coaching Active linkage to recovery communities Rapid access back to treatment where necessary	N/A					
Wiebe et al. (2018)	Weekly attendance of on or off‐campus 12‐step meetings	South‐western U.S. University, USA	Quantitative, exploratory	Undergraduate students in recovery programme for substance abuse	To determine the daily use and factor structure of the 12 steps including the intrapersonal predictors and moderators of 12‐step use	Exploratory factor analysis for factor structure Multilevel models to examine day level and person‐level predictors Robust weighted least squares estimator	Analyses results produced two factors: Everyday steps that comprised surrender and maintenance steps, and action steps	1	0	1	1	1	4
Wong, Zhuang, and Ng (2020)	The intervention group received individual counselling following an integrated cognitive‐behavioural therapy (ICBT) The comparison group received TAU individual counselling	Hong Kong	Quasi‐experimental matched‐pairs comparison design	Youth drug abusers (aged 11–35 years)	To develop and test an ICBT model for helping youth with drug abuse problems in the community setting	Multilevel models A multiple mediational analyses using PROCESS were employed (statistical macro for conducting path and mediation analyses)	There were significantly high quality of life and coping strategies reported by the participants in the ICBT compared to the TAU group. These effects were sustained at the 4‐month follow‐up	1	1	1	1	1	5
You et al. (2020)	10‐week family‐oriented therapy programme	Chang Gung Memorial Hospital, Taiwan	Quantitative nonrandomised (the study was conducted in a noncomparative, nonrandomised manner)	Young patients aged between 12 and 18 years of age with substance use problems	To engage participants in a 10‐week group motivation‐enhanced psychotherapy, with the goal to encourage patients to stop using drugs	Questionnaires Statistical software package SPSS, version 21.0 (SPSS Inc., Chicago, IL, USA)	Patients had better behavioural problems during the treatment programme. The results predicted a lesser likelihood of substance use relapse in the subsequent 5 years	1	1	1	1	1	5

*Note:* N/A, Not applicable (i.e., the use of the MMAT tool does not apply to documents from grey literature and reviews).

**TABLE 2:** Themes and sub‐themes on the prevention of SUR.

Themes	Subthemes
Continuing care	Posttreatment check‐ups Tracking and assessment Linkages to treatment Engagement and retention Use of recovery support services
2 Technology‐mediated recovery management interventions	Mobile aftercare interventions Internet‐based relapse prevention
3 Relapse prevention/recovery promotion through developmentally engaging activities	Physical activities Self‐monitoring Educational activities that promote literacy on substance use

### 2.5 Ethical Considerations

This article adhered to all ethical standards for research without direct contact with human or animal subjects. The article is a component of a larger study and aims to guide the development of a substance use relapse prevention programme for the youth in Botswana. The study has the ethical approval of the Institutional Review Board of North‐West University (Ethics number: NWU‐00174‐23‐S1) and permission from the Ministry of Health IRB (Ref: HPRD: 6/14/1). Permission to conduct the study was also solicited from Sbrana Psychiatric Hospital Institutional Review Board (Ref: 4/2/2 III (65)).

## 3 Findings

**FIGURE 2:**
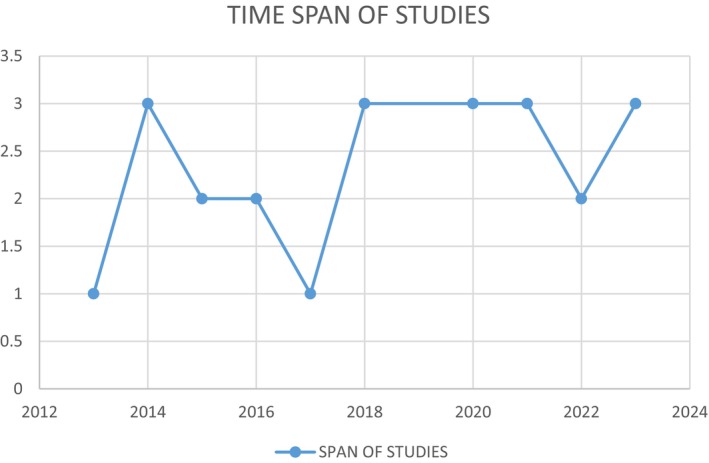
Time span for the selected studies.

Twenty‐six articles were selected using the identified search strategy based on keywords (‘substance OR drug’; AND ‘use OR abuse OR misuse’; ‘relapse prevention OR addiction management’ AND programme OR check‐ups' AND ‘youth OR young adults OR young people’). The review has been reported using the Preferred Reporting Items for Systematic Reviews and Data Analysis. The PRISMA flow chart is an internationally recognised diagram that provides a visual summary of the process used to include and exclude studies in systematic reviews (Page et al. 2021). The PRISMA flow diagram (Figure 1) summarises the search and screening processes used to select the articles. From the 26 articles, 23 were peer reviewed articles and 3 were grey literature. The 23 articles used in this review spanned the period from 2013 to 2024 with most studies conducted between 2017 and 2023 (Figure 2). The studies were conducted in six countries, and more than half of the selected studies were conducted in USA (*n* = 12). Other studies were conducted in South Africa (*n* = 2), Taiwan (*n* = 2), Australia (*n* = 2), Hong Kong (*n* = 2) and Switzerland (*n* = 1). The majority of the studies were randomised controlled trials (RCT) (*n* = 13) followed by quantitative studies (*n* = 4). Mixed methods, qualitative and literature review were each two (*n* = 6) and three (*n* = 3) from grey literature. The details and characteristics of each study are provided in Table 1.

### 3.1 Theme 1: Continuing Care

One of the main themes was continuing care. The initiation and sustenance of behavioural modifications in SUR prevention are discrete procedures that are regulated by certain circumstances (NIDA 2018). Accordingly, various authors concur that to keep young people from relapsing, it is necessary to provide ongoing care after release from the hospital to preserve the progress made during an admission (Bowen et al. 2014; Gonzales, Douglas Anglin, and Glik 2014; ISSUP 2020; Mpanza, Govender, and Voce 2022; NIDA 2018; Scott et al. 2018). The review also demonstrates that the aftercare programme (continued care) should not last for less than 3 months to achieve relapse prevention (Van der Westhuizen 2015). Check‐ups following treatment, monitoring and evaluation, connections to therapy, engagement and retention and utilisation of recovery support programmes are all examples of continuing care.

#### 3.1.1 Posttreatment Check‐Ups

According to the review's findings, posttreatment check‐ups, which are a component of continuing care or recovery management care (RMC), are beneficial for society's financial health and play a significant role in preventing substance use relapses (Dennis, Scott, and Laudet 2014; Gonzales‐Castaneda et al. 2022; NIDA 2018; WHO‐UNODC 2020). Dennis, Scott, and Laudet (2014) state that posttreatment check‐ups are routine, ongoing assessments of substance users to identify relapses early and take appropriate action. According to the literature, posttreatment check‐ups can be conducted through programmes that involve in‐person interactions with youth substance users (YSUs) to strengthen their behavioural and cognitive abilities so they can withstand relapse temptations (Tam, Shik, and Lam 2016; ISSUP 2020). While YSUs are aware of where to turn for support, their biggest obstacle is finding the will to ask for it or to know when to do so (Debenham et al. 2020). To enable young people to participate in health‐seeking behaviour at an early age, the relapse prevention programme with posttreatment check‐ups would, thus, concentrate on increasing their motivation and setting positive, realistic health goals (Debenham et al. 2020; NIDA 2018; Trudeau et al. 2017; Wang et al. 2018).

Nonetheless, the results of this review make it clear that YSUs require assistance in becoming conscious of their cravings and triggers, or in identifying the cognitive, emotional and behavioural dysfunctions that cause them to relapse into substance use, as part of posttreatment check‐ups (Bowen et al. 2014; NIDA 2018). Studies agree that one of the best strategies for treating individuals with substance use disorders is cognitive behavioural therapy (CBT) (Bowen et al. 2014; NIDA 2018; Wong, Zhuang, and Ng 2020). Thus, behavioural and cognitive strategies—either as individual or group therapies—are essential to reducing the likelihood of relapse and easing the cognitive dissonance that often follows in young people's positive behaviour change (Debenham et al. 2020; Tam, Shik, and Lam 2016). Therefore, developing skills to manage urges and postpone gratification, motivation for change, problem‐solving abilities and the ability to engage in nonchemical substance use activities may be among the areas to be addressed at check‐ups (NIDA 2018; WHO‐UNODC 2020; Van der Westhuizen 2015; You et al. 2020).

#### 3.1.2 Tracking and Assessment

The results of this review indicate that monitoring is part of continuous care that requires both assessment and tracking (Dennis, Scott, and Laudet 2014; Karno et al. 2021; NIDA 2018; Scott et al. 2023, 2018). YSUs are proactively tracked and screened (assessed) for early indications of issues, given incentives, and assisted in negotiating access for re‐entry into treatment, among other various functions of continuing care (ISSUP 2020; Scott et al. 2018). The recovery management and relapse prevention manual, also known as the Universal Treatment Curriculum for Substance Use Disorders (UTC), demonstrates the significance of tracking and the proactive steps taken to stay in touch with substance users after they are discharged from treatment (ISSUP 2020).

Fieldwork, phone calls, mailings and other methods are all combined in the tracking process (ISSUP 2020). After that, the drug user is evaluated to rule out SUR and choose the best course of action. To improve outcomes, YSU assessments must be carried out quarterly as part of monitoring for approximately 4 years after discharge (ISSUP 2020; Scott et al. 2018). Consequently, monitoring and evaluation determine whether an individual with a substance use disorder has resumed substance use and allow for intervention to prevent a relapse or lessen its effects (ISSUP 2020).

#### 3.1.3 Linkages to Treatment

During assessment, some YSUs may have relapsed or be on the verge of a relapse. Thus, YSUs in primary care settings require an assertive and robust linkage model that can effectively increase their participation in SUD treatment, leading to significantly greater reductions in drug use (Scott et al. 2018). In situations where relapse is imminent, linking YSUs back to treatment is necessary, thus increasing re‐entry into treatment before a relapse can occur (Dennis, Scott, and Laudet 2014; Karno et al. 2021). Literature provides overwhelming evidence of the effectiveness and value of ‘referral back to treatment’ to prevent relapses to substances (NIDA 2018). In the cases where relapse is imminent, suspected or real, individuals are linked back to inpatient treatment, residential services, SUD outpatient services or community support services to prevent a relapse or minimise its impact (ISSUP 2020).

#### 3.1.4 Engagement and Retention

The literature (NIDA 2018; Scott et al. 2023, 2018) also emphasises the significance of engagement and retention as a component of ongoing treatment to prevent SUR. The engagement component aims to avoid the common roadblocks found throughout the admissions process, allowing substance users to more easily ‘show’ up for treatment following evaluation and referral (ISSUP 2020). To increase the possibility that YSUs will attend treatment after assessment, the engagement process includes putting tactics in place to boost motivation for re‐entry into treatment, expediting the admissions process, and overcoming some of the admissions course's drawbacks (ISSUP 2020; NIDA 2018). After a successful engagement process with the willingness of the YSU to show up to treatment, the primary healthcare linkage manager (focal person) must continue to maintain contact and follow‐up with the substance user to ensure that motivation to participate in treatment is maintained to aid retention (ISSUP 2020; Scott et al. 2018). The linkage manager must provide assistance to overcome any environmental or organisational barriers to retention (Scott et al. 2018).

According to the review's conclusions, the retention component of relapse prevention aims to keep substance users in treatment for a minimum of 14 days, either through inpatient or residential treatment, or 14 sessions of outpatient therapy (ISSUP 2020). To minimise the harm associated with relapse or to sustain substance abstinence, engagement and retention methods are crucial (Scott et al. 2018). The treatment personnel must inform the linkage manager if the YSU makes threats to stop treatment or misses an outpatient visit. The linkage manager then sets up an intervention to encourage re‐engagement in treatment (Scott et al. 2018). Mitchell et al. (2021) emphasised the need for appropriate pharmaceutical treatment postdischarge to prevent relapses and sustain retention to treatment.

#### 3.1.5 Use of Recovery Support Services

According to the review, recovery support services are necessary and encourage healthy behaviours after completing a treatment programme to reduce the risk of a relapse (ISSUP 2020; Mpanza, Govender, and Voce 2022; NIDA 2018). Linking YSUs to recovery support services is crucial as part of reintegration (Van der Westhuizen 2015). The findings of this review indicate that reintegration can be social (social‐reintegration) in which YSUs are assisted to re‐establish themselves into the community through networking with families, schools, churches and support groups (NIDA 2018; WHO‐UNODC 2020). Mpanza, Govender, and Voce (2022) emphasise the need for recovery support services to comprehensively reintegrate people with substance use problems into their families, workplaces and communities. According to the foregoing author, the goal of recovery support services is to provide tools and resources to sustain the recovery of YSUs after the formal treatment programme has been completed.

Additionally, it is evident from this review that participation in recovery support services with more rewarding activities is a key predictor of long‐term recovery (Mpanza, Govender, and Voce 2022). Precisely, the use of recovery support services that aid social integration and participation in conventional pursuits, such as alcohol‐free recreational/art‐based activities and linkages to job placement programmes, is associated with a higher prospect of stable remission and recovery (Mpanza, Govender, and Voce 2022; Tam, Shik, and Lam 2016). Furthermore, the review shows that engagement in peer support groups such as the 12‐step programme is associated with recovery because it provides a conducive environment for establishing positive social relationships, a space for learning new skills and information on health and employment (Mpanza, Govender, and Voce 2022; NIDA 2018). However, it is worth noting from this review that availing resources for recovery support services such as rehabilitation centres in communities is a crucial strategy that facilitates the prevention of relapses and offers an opportunity for the YSUs to effectively and sufficiently address their substance use problems (WHO‐UNODC 2020).

### 3.2 Theme 2: Technology‐Mediated Recovery Management Interventions

One of the themes from the review was technology‐mediated aftercare intervention (TMAI). The use of TMAI, such as mobile aftercare interventions and internet‐based relapse prevention, plays a critical role in preventing relapses among youths either as a standalone or as an adjunct to face‐to‐face interventions.

#### 3.2.1 Mobile Aftercare Interventions

Mobile aftercare intervention is one of the subthemes that emerged in this review under technology‐mediated management interventions. Mobile aftercare interventions can be delivered through smartphone applications or general text messages (SMS) (Gonzales et al. 2014; Gonzales‐Castaneda et al. 2022; Guarino et al. 2016; Haug et al. 2023). From the results of the review, practitioners can continue offering care through text messages to motivate the YSUs to engage in recovery behaviours and to continue making positive life changes (Gonzales, Douglas Anglin, and Glik 2014). Furthermore, Gonzales, Douglas Anglin, and Glik (2014) found that the mobile texting intervention buffered the tendency towards relapse and significantly reduced the severity of substance use among youth who received the aftercare mobile texting intervention compared to the youth in the standard aftercare control condition. Finally, the findings of this review indicate that mobile texting aftercare programme offers several advantages that include personalised and targeted engagement, enhanced assessment and monitoring, increased convenience and better flexibility of service delivery (Gonzales, Douglas Anglin, and Glik 2014).

#### 3.2.2 Internet‐Based Relapse Prevention

Another subtheme that emerged in the review was internet‐based relapse prevention. Internet‐based relapse prevention programming is reported to be a promising modality to enhance treatment gains and recovery (relapse prevention), though Trudeau et al. (2017) argue that it lacks a strong evidence base (Debenham et al. 2020; Dennis, Scott, and Laudet 2014). However, the findings of this review show that digital or internet‐based health interventions can offer frequent monitoring of YSUs and address some of the barriers to health delivery, such as the fear of stigma associated with face‐to‐face interventions (Dennis, Scott, and Laudet 2014; Guarino et al. 2016). Thus, internet‐based interventions can positively impact the provision of substance use prevention or harm reduction initiatives (Debenham et al. 2020; Dennis, Scott, and Laudet 2014; Guarino et al. 2016; Haug et al. 2023; Trudeau et al. 2017). Moreover, the review reveals that internet‐based smartphone applications can be used for engagement, personalised feedback and to provide digital programme modules to be carried out over a time frame (Guarino et al. 2016; Haug et al. 2023).

### 3.3 Theme 3: Relapse Prevention/Recovery Promotion Through Developmentally Engaging Activities

The results of this review indicate the need for relapse prevention through developmentally engaging activities. This involves engaging YSUs on undertakings that have the potential to aid their personal growth and development while also serving as a deterrent to substance use and SUR (Gonzales et al. 2013; ISSUP 2020). Three subthemes were derived from the review: physical activities, educational activities that promote literacy on substance use and self‐monitoring strategies.

#### 3.3.1 Physical Activities

Evidence from the review shows that physical activities, for example, exercise programmes are efficacious in supporting substance use relapse prevention among the youth, especially as a complement to cognitive‐behavioural/lifestyle interventions (Furzer et al. 2021; Gonzales et al. 2013; NIDA 2018; Wong, Zhuang, and Ng 2020). This review demonstrates that the youth who engage in a substance use programme that has physical activities (e.g., exercises) develop enhanced self‐esteem, high relapse prevention efficacy, increased levels of exercise enjoyment and better physical health perceptions (Furzer et al. 2021; Mpanza, Govender, and Voce 2022). However, behaviour change for the youth requires joyful activities (recreational, art‐based, etc.) that can replace substances (Tam, Shik, and Lam 2016; Van der Westhuizen 2015).

#### 3.3.2 Self‐Monitoring

Additionally, the review shows that YSUs need to be empowered with self‐monitoring strategies, for example, use of a diary or technology‐mediated self‐monitoring interventions (Dennis, Scott, and Laudet 2014; Gonzales et al. 2014; Haug et al. 2023; NIDA 2018; Wiebe et al. 2018). Basically, self‐monitoring activities as a relapse prevention and recovery promotion strategies have been found to assist the YSUs to self‐regulate (self‐control) specific and critical areas of their lives associated with relapse following inpatient treatment (Gonzales, Douglas Anglin, and Glik 2014; Guarino et al. 2016). Moreover, the self‐monitoring reports are important to assess for discordance between the self‐report data and sensitivity analyses (urinalysis) to compare if the self‐report of a ‘no recent use’ is supported by substance‐negative urinalysis (Karno et al. 2021).

#### 3.3.3 Educational Activities That Promote Literacy on Substance Use

According to some sources of this review, YSUs need to be psycho‐educated on key neuroscience principles to build their understanding of the brain functions, the impact of substances on the brain and substance use relapses (Debenham et al. 2020; Van der Westhuizen 2015). In a study by Debenham et al. (2020), YSUs who were provided with psychoeducation on neuroscience and the effects of drugs on the brain content found it interesting and these facts made it more believable, which can motivate them to quit and abstain. The psycho‐education message has to be delivered in a creative way to stimulate interest in the YSUs, for example, a combination of art‐based and cognitive‐behavioural‐based relapse prevention groups (Debenham et al. 2020; Tam, Shik, and Lam 2016).

## 4 Discussion

Youth substance use poses a significant problem since it causes dependency and makes it harder to stop using drugs later in life. Even after extended periods of abstinence, young people who begin using substances and attempt to stop generally relapse (Arora et al. 2015). Nevertheless, there is a paucity of empirical data about youth substance use relapse prevention programmes. However, the existing programmes have shown to be successful in preventing SUR and enhancing YSUs' general well‐being. This is especially true in industrialised nations where several relapse prevention initiatives have been created and evaluated for effectiveness.

Surprisingly, this analysis shows that many nations in sub‐Saharan Africa lack SUR preventive programmes, even though substance use is more widespread in this subregion. Consequently, more effort is invested in managing short‐term addiction crises and offering limited aftercare programmes (Saba, Weir, and Aceves‐Martins 2021). Recurring relapses and readmissions are, thus, a common result of such brief recovery therapies and the absence of SUR preventive programmes. While there are many different SUR prevention programmes, this review demonstrates that the most prevalent and effective are cognitive‐behavioural‐based programmes; nevertheless, other strategies, like mindfulness‐based relapse prevention programmes, are also gaining traction.

Most of the authors in our review approached relapse and its prevention in a contextual manner, viewing cognitive and environmental processes as proximal antecedents of relapse. This perspective, however, is different from the one that links relapse to internal causes, such as desires, and views cravings as a sign of an underlying illness (Hendershot et al. 2011). According to Mitchell et al. (2021), the latter of the two perspectives suggests the necessity of pharmaceutical treatment for relapse prevention. The author highlights the importance of receiving proper pharmacological treatment after discharge to maintain treatment retention and prevent relapses.

Regarding the use of medicines for relapse prevention after discharge, the literature is, nevertheless, ambiguous. For example, in contrast to the American Academy of Paediatrics recommendations, the U.S. treatment system prioritises nonmedication treatment for young people (Mitchell et al. 2021). However, most of the programmes in our research focused on providing psychosocial support, like ongoing care to avoid SUR. Sustaining treatment beyond in‐patient discharge is essential for preventing youth substance use relapse (Gonzales et al. 2014; ISSUP 2020; Mpanza, Govender, and Voce 2022). Face‐to‐face interactions can be used to provide ongoing care. It can be utilised to equip YSUs with the abilities to recognise the cognitive, emotional and behavioural dysfunctions that lead to relapse and to resist relapse temptations (ISSUP 2020; Tam, Shik, and Lam 2016). Health professionals in charge of the SUR prevention programmes ought to inspire YSUs to make changes and develop their capacity for problem‐solving and postponing pleasure (NIDA 2018; WHO‐UNODC 2020; Van der Westhuizen 2015). Nonetheless, Van der Westhuizen (2015) reported that a successful SUR prevention programme must be provided for a minimum of 3 months and ought to be sufficiently potent to assist the YSUs in fending off a relapse. People in charge of the SUR prevention programme must keep an eye on and sustain positive behaviour change during the continuing care phase to avoid a relapse. Therefore, the focus should be on strengthening the YSUs' ability to resist a potential relapse and connecting them even more to the resources that are out there for help.

According to the review's findings, connecting substance users with recovery‐focused programmes is essential for their reintegration and support (ISSUP 2020; Van der Westhuizen 2015). Through networking with families, schools, churches and support groups, YSUs are helped to re‐establish themselves into the community during reintegration to promote sustainable rehabilitation (WHO‐UNODC 2020; Mpanza, Govender, and Voce 2022; Teko, Goliath, and Abdulla 2023). Research suggests that social integration support services, job/vocational placement programmes and participation in engaging activities all increase the likelihood of successful recovery and prevent relapses (Gonzales et al. 2013; Mpanza, Govender, and Voce 2022; Tam, Shik, and Lam 2016). Moreover, residential care and rehabilitation facilities are crucial in offering a variety of recovery support programmes that can adequately and successfully treat substance use disorders in individuals who are referred back to them for treatment (Dennis, Scott, and Laudet 2014; Scott et al. 2023; WHO‐UNODC 2020).

The importance and efficacy of technology‐mediated SUR prevention programmes are supported by a small but increasing body of research, particularly when combined with more conventional, in‐person therapy approaches. According to our findings, services for young people who require support with substance use should be tailored to their individual needs. Gonzales‐Castaneda et al. (2022) argue that due to their familiarity with technology, young people can be considered ‘digital natives’. Therefore, to effectively and significantly reach out to young people, technology can provide targeted, tailored interventions and links to therapy as an auxiliary to in‐person interventions.

Technology‐mediated aftercare interventions (TMAI) can be provided by text message, internet‐based digital health care or smartphone applications (Gonzales, Douglas Anglin, and Glik 2014). The linkage manager or focal person in charge of the SUR prevention programme can provide individualised care through the use of text messages or online resources if the usage of TMAI is approved. TMAI can minimise the risk of relapses through active participation in technological recovery‐oriented activities that foster positive life changes. Both personalised feedback and engagement are enhanced through TMAI. For example, YSUs can receive text messages on how to avoid a relapse, substance abuse education, daily self‐monitoring, available community support resources, and they can even participate in digital programme modules (Dennis, Scott, and Laudet 2014; Gonzales, Douglas Anglin, and Glik 2014).

Programmes for the prevention of SUR in youth also need to encourage mental and physical growth (Furzer et al. 2021; ISSUP 2020). To replace the hedonic experience of substances, the programme activities need to be pleasurable (Furzer et al. 2021). According to our research, these kinds of activities ought to promote individual development and act as a disincentive to SUR (Gonzales et al. 2013; ISSUP 2020). Exercise programmes are effective in preventing youth substance use relapses, according to a body of research, especially when they are implemented in conjunction with cognitive‐behavioural/lifestyle change interventions (Furzer et al. 2021; Wong, Zhuang, and Ng 2020). It is necessary to replace the hedonistic experience of drugs with such joyful activities if young people are to have better chances of changing their behaviour (Furzer et al. 2021). Engaging in physical exercise has been shown to improve perceptions of physical health and self‐esteem (Furzer et al. 2021).

However, educational initiatives that promote literacy around substance use and SUR must be combined with physical activities (Van der Westhuizen 2015). YSUs should receive psychoeducation on the fundamental neurobiological concepts underlying habit and addictive behaviours in order to achieve this goal (Debenham et al. 2020). According to our review, youngsters find knowledge about brain processes and their connection to substance use and relapse to be motivating while trying to quit drugs (Debenham et al. 2020; Van der Westhuizen 2015). Like joyful activities, the psycho‐education message needs to be presented in an original manner to pique certain young people's interest (Debenham et al. 2020; Tam, Shik, and Lam 2016). Additionally, self‐monitoring techniques can be used to psycho‐educate YSUs (Gonzales et al. 2014). Practitioners can evaluate whether there is a discrepancy between the self‐report data and sensitivity analysis (urinalysis) using the self‐monitoring reports that they generate (Karno et al. 2021). A drug‐negative test must substantiate a self‐report that excludes any recent substance usage.

### 4.1 Strengths and Limitations of the Review

With the use of this integrative literature review, which offered a strong and practical methodology, a thorough understanding of youth substance use relapse prevention was obtained by integrating the research findings from various study designs. This is the first review on the subject that we are aware of. Nonetheless, this review has certain methodological shortcomings. First, there was bias in the language. The publications that were written in English were the subject of the review. As a result, it is possible that the reviewers overlooked important and pertinent material written in another language. Second, it is possible that some important publications that were not indexed by the chosen databases escaped the reviewers' notice.

Therefore, to reduce this risk, all pertinent studies were found by doing an article search utilising interchangeable or alternative key terms, such as ‘substance OR drug’ and ‘use OR abuse OR misuse’. However, selection bias would have also happened as a result of human error brought on by handling a large number of search results because of the high search sensitivity. However, the reviewers took precautions by reviewing the search results again and screening again to make sure certain important items were not overlooked.

### 4.2 Strengths and Limitations of the Literature

The information gleaned from this analysis contributes to the expanding body of research on youth substance use relapse prevention programmes. The review provides some insight into the crucial steps required to create the SUR preventive programme's preventative tactics. The study makes it abundantly evident that the youth's programme entails ongoing evaluations, supervision and re‐intervention to either lessen the severity of a relapse or maybe re‐enter treatment before it happens. Nonetheless, there remained a paucity of research on youth relapse prevention programmes, particularly in Africa. Furthermore, the majority of the review's conclusions focus on American situations, which can lack the qualities of regional contexts. The review also included grey literature. Despite the lack of peer review, the grey literature and material can provide valuable information to address the study topic (Adams et al. 2016). Evidence syntheses on SUR prevention programmes for young people included information from grey literature and other sources that were deemed reliable and pertinent to address the study topic.

### 4.3 Implication for Practice

In the community, mental health workers such as psychiatric nurses provide care and assist in the rehabilitation of individuals with SUDs. They must comprehend how substance use relapse can be prevented, particularly among the young, since they oversee substance use prevention programmes and SUR prevention programmes (tertiary prevention). Particularly in Africa, where mental health professionals such as psychiatric nurses are sent to serve rural communities as the only mental health professionals in their catchment areas, they play a crucial role in the provision of mental health care. Therefore, mental health workers must comprehend the components of a youth substance use relapse prevention programme. The review's findings are critical to the field of mental health and psychiatry, and they are released at a critical juncture as mental health professionals around the world struggle to find effective ways to reduce drug‐related relapses that impede successful recovery. The results hold the potential to offer well‐informed and comprehensive preventive care, ending the never‐ending cycle of treating recurrent cases of substance use.

## 5 Conclusion

The prevention of substance use relapse in young people with substance use disorders was the main emphasis of this review. Youth SUR prevention must be thorough and realistic enough to touch all facets of their lives. As a result, the youth SUR prevention programme needs to be comprehensive, sensitive to their needs and supportive of partnerships with families, community support agencies, and so on. The review emphasised important components of a successful substance use programme, including ongoing follow‐ups after treatment and evaluations to identify relapses and take immediate action. The evaluations and check‐ups provide connections for harm reduction in the event of a relapse or for re‐entry into treatment before a relapse. In addition, the YSUs are equipped with the knowledge and abilities to resist psycho‐social influences that may cause them to relapse.

## Author Contributions

Four authors contributed to this manuscript submitted for review. W.G. conceptualised the manuscript as part of his PhD study. The three study supervisors M.M.M., G.P.K. and L.S. guided the final critical revisions and editing of the manuscript. The article was proofread by all authors before submission.

## Conflicts of Interest

The authors declare no conflicts of interest.

## Relevance Statement

This review identifies substance use as a serious problem that affects youth substance users. Though substance use is common among youth, their relapse following abstinence causes a great concern and its prevention is given less attention in many countries. This review identified, synthesised, and presented the findings of all relevant studies on substance use relapse prevention for youth with substance use disorders. Mental health nursing care considers substance use relapse as a serious challenge, and it is important to comprehend the components of a youth substance use relapse prevention programme to prevent substance youth relapse.

## Data Availability

The authors have nothing to report.
